# Mechanical Support in Early Cardiogenic Shock: What Is the Role of Intra-aortic Balloon Counterpulsation?

**DOI:** 10.1007/s11897-020-00480-0

**Published:** 2020-09-01

**Authors:** Jesse R. Kimman, Nicolas M. Van Mieghem, Henrik Endeman, Jasper J. Brugts, Alina A. Constantinescu, Olivier C. Manintveld, Eric A. Dubois, Corstiaan A. den Uil

**Affiliations:** 1 Department of Cardiology, Thorax Center, Erasmus University Medical Center, Doctor Molewaterplein 40, 3015 GD Rotterdam, the Netherlands; 2 Department of Intensive Care Medicine, Erasmus University Medical Center, Rotterdam, the Netherlands

**Keywords:** Intra-aortic balloon counterpulsation, Mechanical circulatory support, Cardiogenic shock, Heart failure

## Abstract

**Purpose of Review:**

We aim to summarize recent insights and provide an up-to-date overview on the role of intra-aortic balloon pump (IABP) counterpulsation in cardiogenic shock (CS).

**Recent Findings:**

In the largest randomized controlled trial (RCT) of patients with CS after acute myocardial infarction (AMICS), IABP did not lower mortality. However, recent data suggest a role for IABP in patients who have persistent ischemia after revascularization. Moreover, in the growing population of CS not caused by acute coronary syndrome (ACS), multiple retrospective studies and one small RCT report on significant hemodynamic improvement following (early) initiation of IABP support, which allowed bridging of most patients to recovery or definitive therapies like heart transplant or a left ventricular assist device (LVAD).

**Summary:**

Routine use of IABP in patients with AMICS is not recommended, but many patients with CS either from ischemic or non-ischemic cause may benefit from IABP at least for hemodynamic improvement in the short term. There is a need for a larger RCT regarding the role of IABP in selected patients with ACS, as well as in patients with non-ACS CS.

## Introduction

Although the use of (percutaneous and non-percutaneous) mechanical circulatory support devices (MCSDs) such as veno-arterial extracorporeal membrane oxygenation (VA-ECMO) has increased considerably last years, intra-aortic balloon pump (IABP) counterpulsation globally remains the most used first-line support in patients with cardiogenic shock (CS) [1, 2]. In this article, we aim to summarize recent insights and provide an up-to-date overview of the use of IABP in patients with CS.

### Technique

IABP is a mechanical support device that consists of a flexible 30–50-cc helium-filled balloon catheter attached to a console that times periodic inflation and deflation according to the cardiac cycle. The distal tip of the balloon should be placed in the descending aorta, approximately 1 cm distal to the origin of the left subclavian artery. The IABP was first placed by surgical cut-down of the femoral artery by Dr. Adrian Kantrowitz in the 1960s. Currently, implantation is usually done by a percutaneous (*Seldinger*) technique via the femoral approach, although surgical insertion in the subclavian artery [3–5, 6•] or percutaneous introduction via the axillary artery [7•] is also possible.

### Hemodynamics

**Fig. 1:**
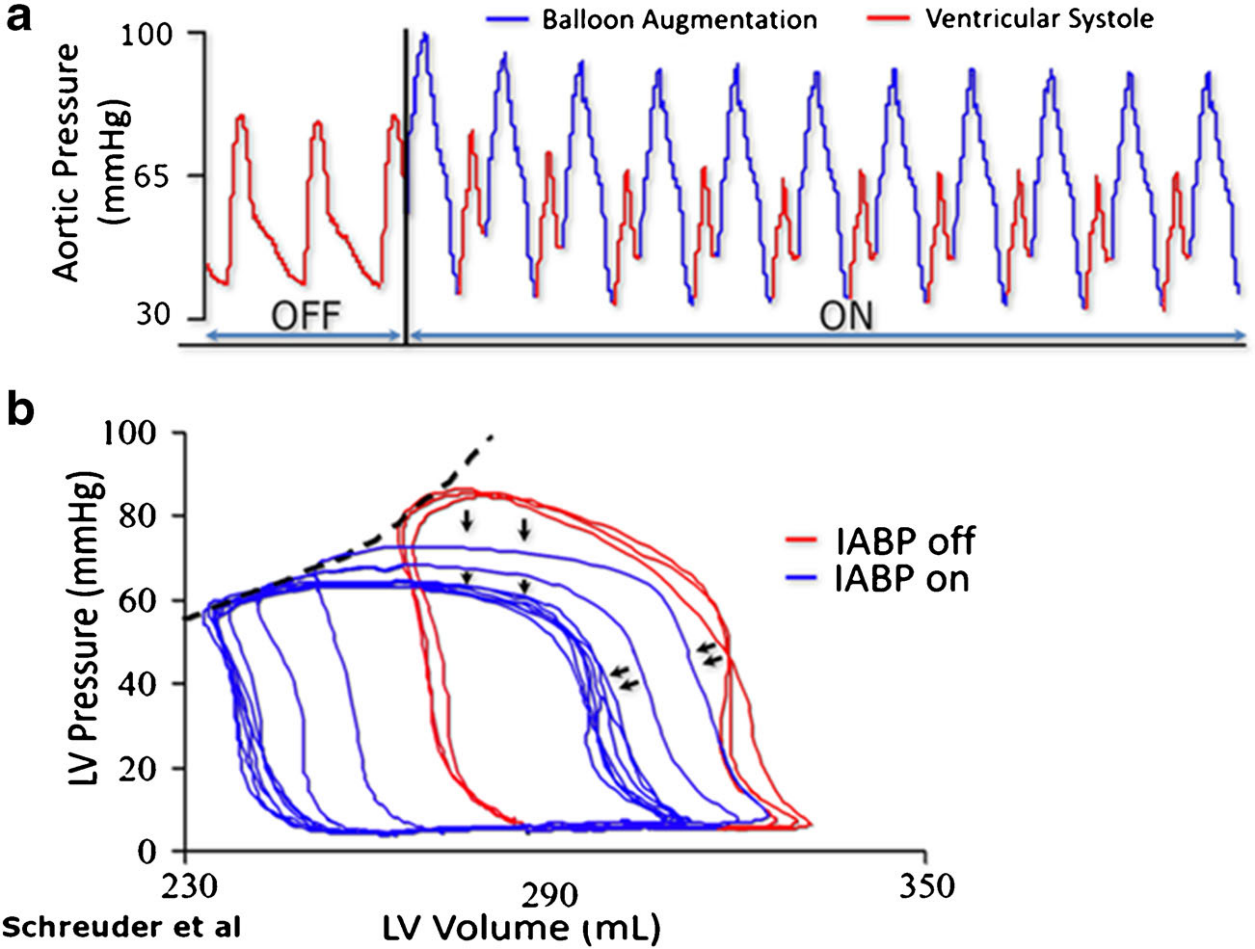
Hemodynamic effects of an IABP in patients with reduced ejection fraction. **a** Immediate effect on aortic pressure curve after initiation of IABP in a patient with 14% ejection fraction. **b** Corresponding pressure-volume loops showing left shift with reduction in systolic pressure, and increased stroke volume. Copied with permission from Bastos et al. [8] and Schreuder et al. [9]

Its physiological effect is dual. By inflating the balloon immediately after aortic valve closure, diastolic and mean arterial pressures rise and coronary perfusion improves. On the other hand, a vacuum effect—caused by rapid deflation of the balloon just before aortic valve opening—provides a reduction in left ventricle afterload and thereby passively augments cardiac output (CO) [8]. The hemodynamic effect will vary based on the clinical setting and the overall stroke volume. In vivo left pressure-volume loops, measured invasively with a conductance catheter, show an acute decrease in left ventricular end-systolic volume by 6%, a decrease in left ventricular end-systolic pressure by 18%, and an increase in stroke volume by 14% (see Fig. 1b) [9]. Left ventricle stroke work is reduced [10]. The primary objectives of the IABP are an increase in myocardial oxygen supply, a decrease in oxygen demand, and optimization of end-organ perfusion [10]. The bedside effects on aortic pressure curves are generally characterized by a decrease in systolic blood pressure, an increase in diastolic blood pressure, and an increase in mean arterial pressure (Fig. 1a) [8]. A reduction in pulmonary capillary wedge pressure (PCWP) and an increase in stroke volume can be measured with right heart catheterization or estimated with echocardiography [8].

### Indications

IABP has been applied in a wide spectrum of indications.

#### Acute Myocardial Infarction Without Shock

* Counterpulsation to Reduce Infarct Size Pre-PCI Acute Myocardial Infarction (CRISP-AMI) was a multicenter randomized controlled trial (RCT) that showed no reduction in infarct size or mortality by a strategy of percutaneous coronary intervention (PCI) with prophylactic IABP support versus PCI alone in 337 patients with anterior ST-elevation myocardial infarction (STEMI) without CS [11]. Nine percent of the patients in the PCI group crossed over to rescue IABP therapy. However, there was a significant difference in the exploratory composite end point of time to death, shock, or new or worsening heart failure (HF) (*P* = 0.03), which was solely driven by the development of shock in patients after PCI.

* In 2015, a meta-analysis to assess IABP efficacy in AMI included 12 RCTs containing a total of 2123 patients [12]. The authors concluded that IABP did not have any statistically significant effect on mortality.

* Recently, Van Nunen and colleagues evaluated the effect of the IABP in 100 patients with large STEMI complicated by persistent ischemia (defined by < 50% of ST-elevation resolution after PCI) [13]. Placement of IABP in this selected group resulted in more frequent ST-elevation resolution (73 ± 17%) compared with the control group (56 ± 26%; *P* < 0.01), after a mean of 3 h. The composite end point of death, necessity of left ventricular assist device (LVAD) implantation, or re-admission for HF within 6 months was numerically lower in the IABP group compared with the control group. The authors found no significant difference in infarct size.

#### High-Risk Percutaneous Coronary Intervention

* In BCIS-1, a multicenter trial, 301 elective patients with severe coronary artery disease and left ventricular ejection fraction (LVEF) of < 30% were randomized to receive PCI with or without IABP support [14]. Twelve percent of the no-IABP group required bailout IABP therapy. This study was primarily designed to address in-hospital MACCE (a composite end point of death, AMI, further revascularization, and cerebrovascular events) at 28 days, and no difference between the groups was seen. However, all-cause mortality at a median follow-up of 51 months was significantly lower in the planned IABP group vs the PCI alone group (HR 0.66; 95% CI 0.44–0.98; *P* = 0.039).

* In the PROTECT II study, 452 symptomatic patients with complex 3-vessel or unprotected left main or last patent coronary artery disease with a LVEF of ≤ 35% were randomized to hemodynamic support by IABP or Impella 2.5 during non-emergent high-risk PCI [15]. Impella provided better hemodynamic support, which was the secondary outcome measure. There was no significant difference in the primary composite end point of MACCE and device-related adverse events after 30 days. However, there was a significantly better outcome of this composite end point in the Impella group after 90 days in the per-protocol analysis (51% in IABP vs 40% in Impella; *P* = 0.02).

* In a recent meta-analysis of 16 RCTs, prophylactic use of IABP during high-risk PCI was not associated with a decrease in 30-day or 6-month all-cause mortality, re-infarction, stroke/transient ischemic attack (TIA), HF, repeat revascularization, embolization, or arrhythmia [16]. Percutaneous ventricular assist devices (pVADs) were more likely to reduce repeat revascularization but showed an increased risk of bleeding events compared with IABP.

* A retrospective analysis of 21,848 patients who underwent non-emergent PCI requiring mechanical circulatory support showed that patients supported with a pVAD had lower in-hospital mortality compared with IABP, despite the observation that patients in this group had more comorbidities [17]. Patients with pVAD also had lower cardiac, vascular, and respiratory complications and their duration of hospital stay was shorter. After applying propensity score matching, these findings remained significant.

#### Prior to High-Risk Coronary Artery Bypass Graft Surgery

Although some meta-analyses suggest a benefit in mortality and MACCE, the prophylactic pre-operative insertion of IABP in patients undergoing high-risk coronary artery bypass grafting (CABG) remains controversial [18–21].

#### As a Left Ventricular Vent During VA-ECMO Support

In patients with CS requiring VA-ECMO, the concomitant use of IABP is associated with significantly lower mortality, although direct unloading by the concomitant use of a (more expensive) Impella device might be even more effective [22, 23]. However, Impella requires larger vascular access and may be associated with more adverse effects (bleeding, hemolysis, limb ischemia).

#### Mechanical Complications of AMI

A final indication includes mechanical complications of AMI (i.e., ventricular septal rupture, mitral regurgitation, or free wall rupture) as a bridge to surgical repair which is still a class IIa/C recommendation for IABP placement in European guidelines [24, 25].

### Adverse Events

Compared with other MCSDs like micro-axial pVADs (Impella, Abiomed, Danvers, MA; USA) and Tandem Heart (CardiacAssist Inc., Pittsburgh, PA, USA), extracorporeal centrifugal-flow LVAD, and VA-ECMO, complication rates of IABP are low. The reported incidence of adverse events in femoral IABP implantation ranges between 0.9 and 31.1% [26•, 27, 28•, 29, 30••], but these rates also include minor adverse events (e.g., access site hematoma, transient loss of pulsations, or need for blood transfusion). The most frequent device-related complication is (most often reversible) limb ischemia with a roughly estimated incidence of 5% (range from 0.9 to 26.7%) [27, 29, 31]. However, we have to consider that complications may be the result of the CS itself, since the complication rate in IABP supported patients was equal compared with controls in IABP-SHOCK [31]. When the IABP is implanted by an axillary or subclavian approach, the following complications have been reported: malfunction due to kinking, rupture, or migration requiring removal or reposition (15–37%), stroke (0–3%), upper limb ischemia (0–4%), transient brachial plexus injury (0–2%), mesenteric ischemia (0–3%), local vascular complications (0–7%), bacteremia requiring antibiotics (0–9%), and bleeding needing transfusion (0–16%) [4, 5, 7•, 32].

## Recent Insights Regarding the Use of IABP in CS

### Cardiogenic Shock After Acute Myocardial Infarction

While cardiogenic shock following acute myocardial infarction (AMICS) was the main indication for an IABP for many years, the results of the IABP-SHOCK II trial in 2012, the largest IABP trial so far, caused a severe decline in its routine use [2, 33, 34]. In this RCT, 600 patients with AMICS were randomized to IABP or conservative therapy, both including routine revascularization [31]. No difference in all-cause mortality after 30 days was observed. On the other hand, IABP was not associated with increased adverse events like re-infarction, stent thrombosis, bleeding, sepsis, or stroke. In 2015, a meta-analysis of 7 RCTs including 790 patients with AMICS showed similar results of no survival benefit by the routine placement of an IABP in this population [35]. As a consequence of these results, both European and American guideline recommendations were downgraded (ESC: III/B; ACC/AHA: IIb/B) [24, 36, 37]. Because only 13% of patients in the IABP group of the IABP-SHOCK II trial received the IABP before revascularization, a meta-analysis including 1348 patients with AMICS was performed in order to clarify the role of the timing of its placement [38]. However, no difference was seen with respect to short- or long-term (≥ 6 months) survival between patients supported upstream or only after primary PCI. Also, no significant outcome difference in terms of re-infarction, repeat revascularization, stroke, renal failure, and major bleeding was seen.

### IABP vs Impella

It is hypothesized that the Impella device, by direct unloading, may reduce infarct size, particularly when starting pre-PCI in patients with AMICS who are revascularized [39]. Patients with CS who were treated with pVAD (Tandem Heart® or Impella®) had a significantly higher mean arterial pressure and a faster decrease in lactate levels compared with patients treated with IABP [40]. However, in the same meta-analysis including 148 patients, no significant difference in 30-d mortality was seen, whereas bleeding occurred more frequently in patients with pVAD (RR 2.50; *P* < 0.001) [40]. Of note, sample sizes of the 4 RCTs included in this meta-analysis were small. Critics also emphasize that 92% of patients in the latest IMPRESS (IMPella versus IABP REduces mortality in STEMI patients treated with primary PCI in Severe cardiogenic SHOCK) study had been resuscitated from cardiac arrest, resulting in a 46% death rate due to anoxic brain damage [41].

Two important observational studies were recently published. First, Schrage and colleagues retrospectively matched 237 patients with AMICS treated with Impella to an equal number of patients from the IABP-SHOCK II trial treated with medical therapy or IABP [42]. The authors found no significant difference in 30-d all-cause mortality, while severe or life-threatening bleeding and peripheral vascular complications occurred significantly more often in the Impella group. Second, in a large US retrospective study including 1680 propensity-matched paired patients with AMICS undergoing PCI, there was a significantly higher risk of in-hospital death and major bleeding associated with the use of pVADs compared with treatment with IABP (45% vs 34% and 31% vs 16% respectively; P for both < .001) [43]. These findings were remarkable since patients with pVADs were significantly younger and less likely to have STEMI compared with patients treated with IABP.

### Large-Volume IABP May Be Better

In the past decade, a larger-capacity (50-cc) IABP was introduced into clinical practice. Compared with previously used 40-cc IABPs, patients who received a 50-cc IABP showed higher-peak augmented diastolic pressure, higher magnitude of diastolic augmentation, and a greater slope and magnitude of deflation pressure from peak augmented diastolic pressure to reduced aortic end-diastolic pressure [44]. In 50-cc IABP recipients, diastolic pressure and PA occlusion pressure were reduced, and CO, cardiac index, and PA oxygen saturation were increased, while these PA catheter–derived measurements did not significantly change in patients with a 40-cc IABP. The absolute increase in CO was 1.4 ± 1.0 L/min in the 50-cc IABP group versus 0.7 ± 0.9 L/min in the 40-cc IABP group, which represented a relative increase of CO compared with baseline of 40% and 18% respectively (*P* = .08). Fifty cubic centimeters IABP also resulted in a greater systolic unloading and a larger reduction in pulmonary capillary occlusion pressure, compared with 40-cc IABP. The magnitude of systolic unloading correlated directly with the magnitude of diastolic augmentation and inversely with the PA occlusion pressure [44]. Also in later studies, 50-cc IABP caused significant diastolic pressure augmentation (Δ + 42 mmHg), systolic unloading (Δ − 15 mmHg), increased CO (Δ + 1.03 L/min), and decreased cardiac filling pressures in the majority of patients [45, 46].

### Non-ACS Cardiogenic Shock

Although the use of IABP in patients with AMICS is now controversial, 20–70% of all CS is not caused by an ACS [2, 47–49]. This non-ACS CS group (also defined as ADHF-CS: acute decompensated HF with cardiogenic shock) includes acute decompensated chronic HF but also CS as a presentation of de novo HF. Importantly, this group seems to be a different population with regard to age, gender, ventricular function, and ventricular dimensions [2, 47, 49, 50••]. Patients with non-ACS CS also have less atherosclerotic cardiovascular risk factors and are more likely to have chronic kidney disease and pre-existing HF, compared with patients with AMICS [47, 48, 50••]. In contrast to AMICS, the etiology of non-ACS CS is diverse, reaching from temporary cardiac disturbances like arrhythmias (responsive to interventions or even self-limiting) until expressions of end-stage HF without any traceable provoking events. Although the role of IABP in this population remains insufficiently defined, several small uncontrolled studies have been performed in order to elucidate its feasibility in this subgroup. These studies are summarized in Table 1.

**Table 1:** Chronologic overview of recently published studies regarding the use of IABP in non-ACS cardiogenic shock and end-stage chronic heart failure

Author, publication year [reference]	Study design (volume of balloon) « insertion site »	Inclusion criteria/study population	No. of pts treated with IABP^*#*^	Duration of IABP therapy (range)	Effects on hemodynamics, echocardiography and laboratory tests^^^	Clinical outcomes
Norkiene, 2007 [51]	Retrospective, observational (40 cc IABP) « femoral »	Acute decompensated DCM, listed for urgent OHT or LVAD, NYHA 4, MAP < 65, CI < 2, PCWP > 20, refractory to all means of OMT	11	Mean 182 ± 82 h (72 to 360)	MAP ↑; LVEF ↑; CVP ↓	27% recovery; 27% LVAD; 18% OHT; 27% died (2 after IABP removal and 1 after LVAD)
Gjesdal, 2009 [52]	Retrospective (40–50 cc IABP) « femoral »	*IABP*: Terminal HF, IABP as an intended BTT due to clinical deterioration not responding to OMT *Control*: Pts who received OHT in a hemodynamic stable situation (without IABP)	40 (control group: 135)	Mean 21 ± 16 days (3 to 66) from onset IABP to OHT Mean 25 ± 21 days (1 to 49) from IABP to MCS	Creatinine ↓; urea ↓; ASAT and ALAT ↓; bilirubin ↓; sodium ↑; potassium ↓	95% OHT, but 15% needed escalation to ECMO (10%) and LVAD (5%); 5% died (2.5% on IABP and 2.5% on LVAD); equal post-OHT mortality after 30 d, 1 y, and 3 y between IABP and control; post-OHT RHC and TTE variables equal after 30 d and 1 y
Russo, 2012 [5]	Retrospective, observational (size NA) « subclavian »	IABP to support severe decompensated HF while awaiting OHT	17^$^	Mean 17 ± 13 days (3 to 48)	NA	82% OHT; 12% needed escalation to VAD (further outcome unknown); 6% still waiting for OHT; 0% died
Umakanthan, 2012 [32]	Retrospective, observational (size NA) « axillary^&^ »	End-stage HF and failure on or intolerance to inotropes	18	Mean 27 ± 18 days (5 to 63) Median 19 days	CI ↑; mPAP ↓; sPAP ↓; CVP ↓	72% OHT; 28% died (6% despite escalation to LVAD); longest walking distance 5.5× ↑; 1 m survival 89%; 6 m survival 72%
Mizuno, 2014 [53]	Prospective, non-randomized, observational, multicenter cohort (size NA) « femoral »	ADHF who meet the modified Framingham criteria, > 20 y, and considered suitable by the attending physicians; *IABP* vs *control* (without IABP)	123 (control group: 4678)	NA	NA	71% discharged alive; 29% mortality during hospitalization; mean length of hospital stay 48 days
Ntalianis, 2015 [54]	Prospective, unicenter, observational (size NA) « femoral »	End-stage HF, NYHA IV, INTERMACS 1 or 2, despite OMT, severe LV and RV systolic dysfunction, with contra-indications for durable HRT, IABP as prolonged support in order to improve the RV function to recover or regain LVAD candidacy	15	Mean 73 ± 50 days (13 to 155) Median 72 days	RAP ↓; mPAP ↓; CI ↑; RVSWI ↑; PCWP ↓; creatinine ↓; total bilirubin↓; LVEF ↑; RVEDD ↓; Sm ↑	20% recovery (without MCS and all alive/NYHA1 after 6 m); 40% LVAD after a mean of 66 d (reversal of previous contra-indications by IABP); 40% died
Sintek, 2015 [55]	Single-centre, retrospective (mean size 42 cc) « femoral »	Systolic CHF who developed CS refractory to OMT and, INTERMACS 1 or 2, pts. who received LVAD after bridge with IABP	54	Median 2 days for decompensated pts and 3 days for stabilized pts	CI ↑; PCWP ↓; CPI ↑; UP ↑; sPAP ↓ only in subgroup of responders	57% stabilized^*^; 43% decompensated (26% medication increase; 11% escalation to MCS); 17% died
Tanaka, 2016 [4]	Single-centre, retrospective (size 34/40/50 cc) « subclavian^&^ »	Advanced DCHF (clinical diagnosis confirmed by RHC), 56% on inotropes, mean CI 1.9 ± 0.6, as a bridge to definitive HRT	88	Median 21 ± 22 days (4 to 135)	CVP↓; mPAP ↓; PCWP ↓; CI ↑; creatinine ↓	93% of patients LVAD, OHT, or recovery (3.4% with escalation to MCS); 7% died; 96% able to walk > 3×/d and received physical rehabilitation during IABP; TMST ↑
Den Uil, 2017 [56]	Single center, retrospective (50cc IABP) « femoral »	Inotrope-dependent HF with signs of hypoperfusion and tissue hypoxia, INTERMACS 1/2	27	Median 4 days (3 to 9)	MAP ↑; sVO2 ↑; RAP ↓; fb ↓; lactate ↓; sodium ↑	67% successful (26% recovery; 19% LVAD; 22% OHT); 7% escalation to ECMO; 26% died; 30-day survival 67%; 1 y survival 63%
Annamalai, 2017 [10]	Single-centre, prospective (50 cc IABP) « femoral »	Stage D HF, NYHA 3/4, INTERMACS 2/3, inotrope-dependent with persistently low CO, within 48 h of LVAD surgery	10	< 48 h	LVSW ↓; LVESP ↓; DPTI ↑; PAP ↓; myocardial oxygen supply/demand ratio ↑; PVR ↓; CPO ↑	100% successful LVAD
Hsu, 2018 [26•]	Single-centre, retrospective, cohort study (size NA) « femoral »	> 18 y, CS (89% systolic CHF) defined as SBP < 90 for > 30 min with evidence of poor end-organ perfusion or need for inotropic support	74	NA	CI ↑; SVR ↓; HR ↓; SBP ↓; DBP ↓; RAP ↓; PCWP ↓; PAP ↓; LVCPI ↑;	20% recovery; 45% LVAD; 7% OHT; 4% urgent escalation to MCS; 24% died
Morici, 2018 [57]	Bicentre, prospective, phase II study (size NA) « femoral »	≥ 18 y, < 80 y, severe LV dysfunction, SBP < 90, or MAP < 60 after fluid challenge or with RAP > 12 or PCWP > 14 with ≥ 1 sign of ongoing organ hypoperfusion, failure of OMT (88% after failure of inotropes)	17^$^	Median 7 days (IQR 4 to 9)	NA for IABP alone group	12% recovery; 53% LVAD; 12% OHT; 6% escalation to ECMO; 18% died
Fried, 2018 [28•]	Single-centre, retrospective, cohort study (size NA) « femoral except for 1 axillary»	≥ 18 y, ADCHF with CS (CI < 2.2 and SBP < 90 or need for vasoactive medications to maintain this level) (87% on ≥ 1 inotrope and 28% on ≥ 1 vasopressor)	132	Median 96 h (IQR 48 to 144) for entire cohort Median 111 h (IQR 48 to 168) for those who received LVAD or OHT Median 84 h (IQR 44–235) for those with clinical deterioration	CO and CI ↑; mPAP ↓	78% discharged after HRT or recovery; 16% recovery; 52% LVAD; 6% OHT; 8% escalation to other MCS; 18% died; 84% overall 30-d survival
Imamura, 2018 [6•]	Single-centre, retrospective (size NA) « subclavian »	Advanced HF, IABP to treat hemodynamic deterioration (69% on inotropes)	91	Mean 25 ± 20 days; 65% continued IABP support for ≥ 14 days	PCWP ↓; CVP ↓; CI ↑; creatinine ↓; lactate ↑	12% recovery; 69% LVAD or OHT; 4% escalation to other MCS; 9% died
Malick, 2019 [50••]	Single-centre, retrospective, cohort study (size NA) « femoral »	≥ 18 y, ADHF with CS (CI < 2.2 and either SBP < 90 or need for vasoactive medications to achieve this SBP)	132^$^	Median 3 days (IQR 2 to 5)	CO and CI ↑; CPO ↑; CPI ↑; CVP ↓; SVR ↓; mPAP ↓	16% recovery; 62% HRT; 22% died; (8% escalation to MCS of which ½ died and ½ received OHT)
Bhimaraj, 2020 [7•]	Single-centre, retrospective, (size NA) « axillary »	Advanced HF who needed maintenance of hemodynamic support until HRT (71% on inotropes), mean sVO2 54%	195	Median 19 days (IQR 12 to 43), max 169 days	WBC ↓; BUN ↓; bilirubin ↓	68% successful HRT (62% OHT and 7% LVAD); 9% escalation to MCS; 11% IABP removal due to complications; 8% died and 3% IABP removal because of lack of candidacy for HRT

*ACS* acute coronary syndrome, *ADCHF* acute decompensated chronic heart failure, *ADHF* acute decompensated heart failure, *ALAT* alanine aminotransferase, *ASAT* aspartate aminotransferase, *BTT* bridge to transplant, *BUN* blood urea nitrogen, *cc* cubic centimetre, *CHF* chronic heart failure, *CI* cardiac index (in L/min/m^2^), *CO* cardiac output, *CPO* cardiac power output, *CS* cardiogenic shock, *CVP* central venous pressure, *DBP* diastolic blood pressure (in mmHg), *DCHF* decompensated chronic heart failure, *DCM* dilated cardiomyopathy, *DPTI* diastolic pressure time index, *ECMO* extracorporeal membrane oxygenation, *fb* fluid balance, *HF* heart failure, *HR* heart rate, *HRT* heart replacement therapy (conventional cardiac surgery, heart transplant, or LVAD implantation), *IABP* intra-aortic balloon pump, *INTERMACS* Interagency Registry for Mechanically Assisted Circulatory Support profile, *IQR* interquartile range, *LV* left ventricle, *LVAD* left ventricular assist device, *CPI* cardiac power index, *LVEF* left ventricular ejection fraction, *LVESP* left ventricular end-systolic pressure, *LVSW* left ventricle stroke work, *m* month, *MAP* mean arterial pressure (in mmHg), *max* maximum, *MCS* mechanical circulatory support, *mPAP* mean pulmonary artery pressure (in mmHg), *NA* not available, *No*. number, *NYHA* New York Heart Association classification, *OHT* orthotopic heart transplantation, *OMT* optimal medical (drug) therapy including inotropic and/or vasopressive support, *PAP* pulmonary artery pressure (in mmHg), *PCWP* pulmonary capillary wedge pressure (in mmHg), *Pts* patients, *PVR* peripheral vascular resistance, *RAP* right atrial pressure (in mmHg), *RHC* right heart catheterization, *RV* right ventricle, *RVEDD* right ventricle end-diastolic diameter, *RVSWI* right ventricle stroke work index, *SBP* systolic blood pressure (in mmHg), *Sm* tricuspid annular systolic tissue Doppler velocity, *sPAP* systolic pulmonary artery pressure (in mmHg), *sVO2* central venous oxygen saturation, *TMST* two-minute step in place test, *TTE* transthoracic echocardiography, *UP* urinary production, *VAD* ventricular assist device, *WBC* white blood count, *y* year(s)

^*#*^Only studies with ≥ 10 patients were included in this table

^^^Only significant (*P* < 0.05) results are listed

^$^The overall study population also contained patients with AMICS, other indication for IABP than CS, or control patients without IABP but these patients were excluded from this table

^*^Stabilization means that all the following 5 criteria were met: (1) did not need any other form of temporary mechanical support; (2) did not require an increase in dose or number of vasopressor or inotrope support; (3) did not need renal replacement therapy or mechanical ventilation; (4) did not have refractory ventricular arrhythmias; or (5) did not experience worsening metabolic acidosis

^&^Patients first underwent femoral IABP placement to evaluate if any hemodynamic benefit was achieved

A study of particular interest is the one by Malick and colleagues, in which the effect of IABP placement was directly compared between patients with AMICS (*n* = 73; 36%) and those with non-ACS CS (*n* = 132; 64%) [50••]. Baseline characteristics showed that patients with non-ACS CS had significantly higher PAP (mean 38 ± 9 vs 31 ± 8 mmHg), lower LVEF (18 ± 9 vs 30 ± 12%), higher left ventricular end-diastolic dimension (7 ± 1 vs 5 ± 1 cm), higher serum creatinine (1.97 ± 1.06 vs 1.59 ± 1.11 mg/dL), lower serum lactate (2.54 ± 2.50 vs 4.92 ± 4.21 mmol/L), higher PA pulsatility index (2.91 ± 3.35 vs 2.00 ± 1.69), and more vasoactive agents (1.7 ± 1.0 vs 1.4 ± 0.8). Interestingly, patients with non-ACS CS experienced a 5-fold greater CO augmentation compared with patients with AMICS (0.58 ± 0.79 L/min vs 0.12 ± 1.00 L/min; *P* = 0.0009). Patients with non-ACS CS experienced an increase by almost a quarter (24%) of their baseline CO, while the increase in patients with AMICS was only 10% (*P* = 0.02). Systemic vascular resistance decreased significantly in non-ACS CS patients but remained equal in patients with AMICS (*P* < 0.05).

We recently performed the first RCT regarding IABP therapy versus inotropy in the early phase of non-ACS CS [30••]. The population included both de novo and acute on chronic HF patients without signs of acute ischemia. All patients (*n* = 32) had a systolic blood pressure of < 100 mmHg, fluid retention, at least moderate tricuspid valve regurgitation and/or mitral valve regurgitation, a dilated inferior cava vein, high filling pressure, low CO, a neutral or positive fluid balance despite fluid restriction, and high-dose intravenous loop diuretics, together with dysfunction of at least 1 other organ. Sixteen patients were treated with a 50 cc IABP and 16 with inotropes. After 48 h, those treated with IABP had significant higher central venous oxygen saturation (+ 17 vs. + 5%), a better increase in cardiac power output (+ 0.27 vs + 0.09 W/m^2^), lower N-terminal pro B-type natriuretic peptide levels (− 59 vs − 16 ng/L), a more negative cumulative fluid balance (− 3.066 vs − 1.198 L), and a better decrease in dyspnea severity score (− 4 vs − 2). In addition, mean arterial pressure increased more in the IABP group, and mean PAP and PCWP decreased more in the IABP group. Fewer patients in the IABP group ended up with moderate to severe mitral valve regurgitation. Finally, patients treated with an IABP tended to have lower major adverse cardiovascular events (a combined end point of crossover or other escalation of therapy, death, HF, re-hospitalization or TIA/stroke) (38% vs 69%), and mortality at 90 days (25% vs 56%), when compared with the group of patients who were treated by inotropes only.

## Discussion

### Advantages of IABP Compared With Other MCSDs

Although other MSCDs like Impella, Tandem Heart, or VA-ECMO provide more hemodynamic support, (first-line) IABP has multiple advantages. First of all, it is relatively cheap [1] and IABPs are largely available and applicable, also in non-tertiary centers. Insertion of an IABP device is more straightforward and can be performed in the intensive care unit without the need for fluoroscopy. Compared with other devices, IABP placement is associated with fewer adverse events like vascular complications [58] or hemolysis [39]. Although mobilization of patients with femoral IABPs is compromised, placement in the axillary or subclavian artery allows mobilization and early physical rehabilitation [3–5, 6•, 7•]. When the IABP fails or cannot be weaned, rapid escalation is possible to percutaneous MCSDs, VA-ECMO, or advanced HF therapies like durable MCSDs (e.g. LVAD) or orthotopic heart transplant (OHT) [59]. Finally, an IABP is easily removed and the presence of an IABP does not complicate native heart excision in case of bridging to OHT.

### Why Did IABP Not Provide Benefit in AMICS?

The hemodynamic effects of an IABP stand out better with larger balloon size. Several recent studies demonstrate that the use of larger 50-cc balloons resulted in a greater reduction in cardiac filling pressures and increased CO compared with the 40-cc IABPs [44–46]. Unfortunately, 50-cc IABPs were generally not used in the major landmark studies so far, since the 50-cc IABP was only introduced in 2012. Since the number of patients achieving optimal hemodynamic benefit from IABP activation may be < 50% with the older 30–40-cc IABPs, this could potentially have contributed to the failure of previous IABP studies [44].

Although the supposed additional beneficial effect of improved coronary blood flow by IABP would be expected to be extra beneficial for patients with AMICS, IABP-SHOCK II showed no benefit of survival [31]. Several limitations of the IABP-SHOCK II should be mentioned. As discussed previously, most patients were treated with conventional, small-volume IABP-catheters. Besides, 10% of patients in the control group experienced crossover to IABP. Moreover, since almost half of all patients were included after cardiopulmonary resuscitation, a substantial amount might have died due to post-anoxic damage. Finally, a large percentage of patients in this trial were already on vasopressors/inotropes (90%), and thus IABP therapy might have been initiated too late.

Besides the limitations of this study, there are also several possible pathophysiological explanations for the neutral findings of IABP in patients with AMICS. First, ACS-driven (extensive) myocardial damage triggers inflammatory and other systemic responses, which may be insufficiently counter-attacked by an IABP that only passively supports the circulation [37]. Second, the effect of improved coronary blood flow is possibly non-existent in vivo due to intact coronary autoregulation [13]. Hence, Van Nunen and colleagues postulated the hypothesis that IABP only improves coronary blood flow in case of exhausted coronary autoregulation, which was not the case in IABP-SHOCK II, since 90% of the total study population obtained successful reperfusion (i.e., final TIMI flow grade 2 or 3 in the infarct-related artery (IRA)) [13, 31]. Patients with AMI and persistent ischemia despite primary PCI were supposed to have impaired autoregulation and Van Nunen proved that the IABP resulted in more rapid ST-elevation resolution in this subgroup. Also, death, necessity of LVAD implantation, or re-admission for HF tended to occur less frequently after IABP implantation in this subgroup [13]. Hawranek retrospectively evaluated patients with AMICS from the prospective nationwide registry who had unsuccessful PCI (i.e., final TIMI flow grade 0 to 1 in the IRA) [60•]. Although conclusions are limited by its observational design, IABP in this subgroup was associated with lower short-term and 12-month mortality.

### Why Is the Augmentation of Cardiac Output in Patients With Non-ACS CS More Pronounced Than in Patients With AMICS?

Due to improved survival after ACS, the incidence of end-stage HF and non-ACS CS is rising [61]. However, at this time, no large RCTs for the acute mechanical treatment of this subgroup are available [36]. The first (small) RCT showed significant improvement of central venous oxygen saturation, cardiac power output, and urine output by IABP compared with medical therapy [30••]. Baseline hemodynamic parameters were equal to those reported in previous studies on AMICS [62]. Besides, as we show in Table 1, multiple retrospective studies reported that the use of an IABP in non-ACS CS temporarily stabilized hemodynamics and end-organ perfusion and allowed a bridge to recovery of the native cardiac function, decision-making, or more durable heart replacement therapy like OHT and LVAD. The increase of the cardiac index in non-ACS CS ranged from 0.3 to 0.9 L/min/m^2^ [6•, 28•, 32, 50••, 54], and one may imagine that such a (limited) CO augmentation may be sufficient to stabilize patients with chronic HF and CS who are used to have a low CO under stable conditions. Previous studies of patients with AMICS demonstrated less CO augmentation by IABP [62–64], which probably explains the lack of efficacy in (tachycardic) patients suffering from an acute decrease in stroke volume as included in the IABP-SHOCK II trial [31].

Malick et al. also described that the augmentation of CO occurred to a less extent in patients with AMICS [50••]. The exact reasons for the difference in treatment response between non-ACS CS and AMICS remain unclear. One hypothesis is that IABP support depends on the intrinsic contractile reserve [50••, 65]. Although baseline stroke volume may be identical in AMICS versus non-ACS CS [50••], baseline PAP was higher in non-ACS CS. Since low output may be mainly triggered by high filling pressures in non-ACS CS, and the IABP may be more effective in lowering afterload and optimizing renal perfusion in this subgroup, the IABP may function better in a high-volume status rather than in an acutely developed low-flow contractile state. This explanation is supported by Fried’s finding that non-ACS patients with high baseline mean PAP had the greatest CO augmentation by IABP [28•]. Also in Imamura’s study, patients with higher filling pressures were most likely to benefit from IABP support [6•].

### Clinical Outcomes After IABP in Non-ACS CS

The proportion of patients successfully weaned from IABP in CS is significantly lower in patients with STEMI compared with patients with NSTEMI and congestive HF (*P* = 0.04) [66]. In this retrospective analysis, even 97.8% of congestive HF patients were weaned from IABP support [66]. In Thiele’s IABP-SHOCK II trial, only 4% of patients who received an IABP were bridged to durable mechanical circulatory support with good long-term outcome [31], and in most other AMICS studies, the rates of successful bridging to durable heart replacement therapy were unfortunately not reported [59]. As shown in Table 1, many patients with non-ACS CS treated with IABP were successfully bridged to durable heart replacement therapy like LVAD or OHT. In our recently published RCT, non-ACS CS patients treated with IABP were significantly more often bridged to LVAD or OHT compared with patients treated with inotropes (31 vs 0% respectively; *P* < 0.05) [30••]. Recent literature shows that patients with ischemic or non-ischemic heart failure who needed pre-operative IABP have similar short- and long-term survival rates after LVAD implantation (88% and 78% after 3 and 12 months respectively), compared with patients who received LVAD without the need for pre-operative mechanical circulatory support (91% and 82% after 3 and 12 months respectively) [67••]. Also, after OHT, no significant difference in short- or long-term survival post-OHT between pre-OHT IABP and a control group was seen [52]. Unfortunately, most studies looking specifically at IABP in non-ACS CS (Table 1) did not report long-term survival rates.

### Patient Selection

As already mentioned, CS cannot be seen as one single entity, but rather as a wide spectrum of different aetiologies, hemodynamic characteristics, degree of severity, and response to therapy. This heterogeneity is the main reason that estimating the possible effect of IABP in daily clinical practice remains challenging. Even within the non-ACS CS subgroup, part of the patients appeared to be non-responders [28•]. In 60/75 patients who underwent right heart catheterization in the before-mentioned cohort of Visveswaran, CO and cardiac index increased up to 7 L/min and 3.4 L/min/m^2^ respectively, while in the remaining 20% non-responders CO decreased. Remarkably, the mortality rate between responders and non-responders was equal [46]. In Hsu’s study, all patients showed an initial improvement in CO within the first 24 h, but in patients with adverse events, CO declined after 24–48 h post IABP implantation [26•]. Some authors suggest that the IABP is less effective in patients with non-ACS CS and underlying ischemic cardiomyopathy [26•, 30••]. Others showed that patients with too bad left and/or right ventricle function at baseline were less likely to show clinical stabilization after IABP insertion [10, 26•, 28•, 55, 56, 68]. Many other prognostic parameters at baseline have been proposed (e.g., left ventricular end-diastolic pressure, left ventricle end-systolic pressure, end-systolic pressure-volume relationship, dP/dTmax, right atrial pressure, PAP, right atrial pressure to PCWP ratio, PCWP, left ventricular end-diastolic dimension, heart rate, systemic vascular resistance, absence of biventricular failure, and the degree of inflammation and multi-organ dysfunction), but most study populations were small, sometimes data are conflicting, and underlying mechanisms remain insufficiently understood [6•, 7•, 10, 28•, 30••, 44]. Also, the fact that persisting arrhythmias can cause opposite disadvantageous hemodynamic effects in patients with IABP should always be taken into consideration [4, 8].

### What Is the Correct Timing of IABP Placement?

Although recommended as first-line therapy of CS [36], the beneficial effect of intravenous positive inotropes and/or vasopressors is never proven and observational data even point towards increased mortality [69, 70]. Possible deleterious effects can be explained by an increased incidence of arrhythmias and aggravation of myocardial ischemia. Since primary IABP placement showed substantial and fast hemodynamic benefit as compared with inotrope therapy [30••], early IABP implantation might result in better outcomes. In Gul’s study, placement of IABP within 1 h of onset of CS showed remarkably lower mortality compared with delayed implantation (35% vs 49% respectively; *P* < 0.001) [27], suggesting that early IABP placement instead of waiting too long for the possible benefit of inotropes could be beneficial. This is endorsed by the finding that patients who stabilized after IABP were on fewer vasopressors or inotropes in observational studies [28•, 55]. Unfortunately, in the currently available retrospective studies regarding non-ACS CS (Table 1), the timing of IABP insertion and phase of shock is very heterogeneous and sometimes poorly defined. Also in this population, the timing of implantation seems to be a crucial factor, since the time to mechanical support is proportional to the amount of organ preservation. Finally, also the timing of IABP weaning seems to be crucial and is actually poorly defined in previous studies.

### Areas to Be Discovered

Results of randomized trials like the DanGer Shock and ECLS SHOCK are expected to elucidate the effect on LVEF and mortality by respectively Impella CP and ECMO in patients with AMICS. Since IABP might still provide benefit in selected patients with AMICS and unsuccessful revascularization or patients with non-ACS CS, larger RCTs are required to evaluate its effect in those patients. We would recommend hemodynamically guided placement of IABP in those subgroups. Investigators should preferably evaluate not only outcomes like short-term mortality, but also time to reversal of shock, end-organ failure, duration of hospital stay, and long-term mortality and functionality.

## Conclusion

The IABP remains a relatively cheap and easily applicable device with low complication rates that offers sufficient hemodynamic support in many patients and allows direct escalation to more powerful support devices if necessary. Although IABP is already in use for several decades, strong evidence by large RCTs is still lacking. The largest RCT of IABP in patients with AMICS reported no mortality benefit, but recent data suggest that IABP may still be useful in a selected subgroup (patients with persistent ischemia or unsuccessful revascularization). Moreover, IABP was not harmful either and more importantly this trial did not address CS complicating (chronic) HF without ACS. Available evidence suggests that the IABP has a clear beneficial effect on many hemodynamic parameters in this non-ACS CS group, allowing the clinician to, at least temporarily, stabilize the hemodynamic profile. Although further research is required, the IABP in this particular group seems promising. More studies should be performed to better define other subgroups with good IABP response, particularly in an era where alternative MSCDs or VA-ECMO are available.

## Abbreviations

30-d: 30 day
ACC: American College of Cardiology
ACS: Acute coronary syndrome
AHA: American Heart Association
AMI: Acute myocardial infarction
AMICS: Cardiogenic shock after acute myocardial infarction
BCIS-1: Balloon pump–assisted Coronary Intervention Study
CABG: Coronary artery bypass grafting
Cc: Cubic centimeter
CO: Cardiac output
CRISP-AMI: Counterpulsation to Reduce Infarct Size Pre-PCI Acute Myocardial Infarction
CS: Cardiogenic shock
ESC: European Society of Cardiology
HF: Heart failure
IABP: Intra-aortic balloon pump
LVAD: Left ventricular assist device
LVEF: Left ventricular ejection fraction
MACCE: Major adverse cardiac and cerebrovascular events
MCSD: Mechanical circulatory support device
NSTEMI: non ST-elevation myocardial infarction
OHT: Orthotopic heart transplantation
PCI: Percutaneous coronary intervention
PROTECT II: Prospective Multicenter Randomized Trial Comparing IMPELLA to IABP in High-Risk PCI II
PA: Pulmonary artery
PAP: Pulmonary artery pressure
PCI: Percutaneous coronary intervention
PCWP: Pulmonary capillary wedge pressure
pVAD: Percutaneous ventricular assist device
RCT: Randomized controlled trial
STEMI: ST-elevation myocardial infarction
US: United States
TIA: Transient ischemic attack
TIMI: Thrombolysis in myocardial infarction
IRA: Infarct-related artery
VA-ECMO: Veno-arterial extra-corporal membrane oxygenation

## References

[1] Doshi R, Patel K, Decter D, Gupta R, Meraj P (2019). Trends in the utilisation and in-hospital mortality associated with short-term mechanical circulatory support for heart failure with reduced ejection fraction. Heart Lung Circ.

[2] Shah M, Patnaik S, Patel B, Ram P, Garg L, Agarwal M, Agrawal S, Arora S, Patel N, Wald J, Jorde UP (2018). Trends in mechanical circulatory support use and hospital mortality among patients with acute myocardial infarction and non-infarction related cardiogenic shock in the United States. Clin Res Cardiol.

[3] Raman J, Loor G, London M, Jolly N (2010). Subclavian artery access for ambulatory balloon pump insertion. Ann Thorac Surg.

[4] Tanaka A, Tuladhar SM, Onsager D, Asfaw Z, Ota T, Juricek C, Lahart M, Lonchyna VA, Kim G, Fedson S, Sayer G, Uriel N, Jeevanandam V (2015). The subclavian intraaortic balloon pump: a compelling bridge device for advanced heart failure. Ann Thorac Surg.

[5] Russo MJ, Jeevanandam V, Stepney J, Merlo A, Johnson EM, Malyala R, Raman J (2012). Intra-aortic balloon pump inserted through the subclavian artery: a minimally invasive approach to mechanical support in the ambulatory end-stage heart failure patient. J Thorac Cardiovasc Surg.

[6] Imamura T, Juricek C, Nguyen A, Chung B, Rodgers D, Sayer G (2018). Predictors of hemodynamic improvement and stabilization following intraaortic balloon pump implantation in patients with advanced heart failure. J Invasive Cardiol.

[7] Bhimaraj A, Agrawal T, Duran A, Tamimi O, Amione-Guerra J, Trachtenberg B (2020). Percutaneous left axillary artery placement of intra-aortic balloon pump in advanced heart failure patients. JACC Heart Fail.

[8] Bastos MB, Burkhoff D, Maly J, Daemen J, den Uil CA, Ameloot K, Lenzen M, Mahfoud F, Zijlstra F, Schreuder JJ, van Mieghem NM (2020). Invasive left ventricle pressure-volume analysis: overview and practical clinical implications. Eur Heart J.

[9] Schreuder JJ, Maisano F, Donelli A, Jansen JR, Hanlon P, Bovelander J (2005). Beat-to-beat effects of intraaortic balloon pump timing on left ventricular performance in patients with low ejection fraction. Ann Thorac Surg.

[10] Annamalai SK, Buiten L, Esposito ML, Paruchuri V, Mullin A, Breton C, Pedicini R, O’Kelly R, Morine K, Wessler B, Patel AR, Kiernan MS, Karas RH, Kapur NK (2017). Acute hemodynamic effects of intra-aortic balloon counterpulsation pumps in advanced heart failure. J Card Fail.

[11] Patel MR, Smalling RW, Thiele H, Barnhart HX, Zhou Y, Chandra P, Chew D, Cohen M, French J, Perera D, Ohman EM (2011). Intra-aortic balloon counterpulsation and infarct size in patients with acute anterior myocardial infarction without shock: the CRISP AMI randomized trial. JAMA..

[12] Ahmad Y, Sen S, Shun-Shin MJ, Ouyang J, Finegold JA, Al-Lamee RK (2015). Intra-aortic balloon pump therapy for acute myocardial infarction: a meta-analysis. JAMA Intern Med.

[13] van Nunen LX, van 't Veer M, Zimmermann FM, Wijnbergen I, Brueren GRG, Tonino PAL (2020). Intra-aortic balloon pump counterpulsation in extensive myocardial infarction with persistent ischemia: the SEMPER FI pilot study. Catheter Cardiovasc Interv.

[14] Perera D, Stables R, Clayton T, De Silva K, Lumley M, Clack L (2013). Long-term mortality data from the balloon pump-assisted coronary intervention study (BCIS-1): a randomized, controlled trial of elective balloon counterpulsation during high-risk percutaneous coronary intervention. Circulation..

[15] O’Neill WW, Kleiman NS, Moses J, Henriques JP, Dixon S, Massaro J (2012). A prospective, randomized clinical trial of hemodynamic support with Impella 2.5 versus intra-aortic balloon pump in patients undergoing high-risk percutaneous coronary intervention: the PROTECT II study. Circulation..

[16] Shi W, Wang W, Wang K, Huang W (2019). Percutaneous mechanical circulatory support devices in high-risk patients undergoing percutaneous coronary intervention: a meta-analysis of randomized trials. Medicine (Baltimore).

[17] Al-Khadra Y, Alraies MC, Darmoch F, Pacha HM, Soud M, Kaki A (2020). Outcomes of nonemergent percutaneous coronary intervention requiring mechanical circulatory support in patients without cardiogenic shock. Catheter Cardiovasc Interv.

[18] Deppe AC, Weber C, Liakopoulos OJ, Zeriouh M, Slottosch I, Scherner M, Kuhn EW, Choi YH, Wahlers T (2017). Preoperative intra-aortic balloon pump use in high-risk patients prior to coronary artery bypass graft surgery decreases the risk for morbidity and mortality-a meta-analysis of 9,212 patients. J Card Surg.

[19] Rampersad PP, Udell JA, Zawi R, Ouzounian M, Overgaard CB, Sharma V, Rao V, Farkouh ME, Džavík V (2018). Preoperative intraaortic balloon pump improves early outcomes following high-risk coronary artery bypass graft surgery: a meta-analysis of randomized trials and prospective study design. J Invasive Cardiol.

[20] Escutia-Cuevas HH, Suarez-Cuenca JA, Espinoza-Rueda MA, Macedo-Calvillo L, Castro-Gutierrez A, Garcia-Garcia JF, et al. Preoperative use of intra-aortic balloon pump support reduced 30-day mortality in a population with LVEF >35% and high surgical risk after coronary artery bypass graft surgery. Cardiology. 2020;145(5):267–274. 10.1159/000506393 32222708

[21] Ranucci M, Castelvecchio S, Biondi A, de Vincentiis C, Ballotta A, Varrica A, Frigiola A, Menicanti L (2013). A randomized controlled trial of preoperative intra-aortic balloon pump in coronary patients with poor left ventricular function undergoing coronary artery bypass surgery*. Crit Care Med.

[22] Vallabhajosyula S, O’Horo JC, Antharam P, Ananthaneni S, Vallabhajosyula S, Stulak JM (2018). Concomitant intra-aortic balloon pump use in cardiogenic shock requiring veno-arterial extracorporeal membrane oxygenation. Circ Cardiovasc Interv.

[23] Baldetti L, Gramegna M, Beneduce A, Melillo F, Moroni F, Calvo F, Melisurgo G, Ajello S, Fominskiy E, Pappalardo F, Scandroglio AM (2020). Strategies of left ventricular unloading during VA-ECMO support: a network meta-analysis. Int J Cardiol.

[24] Ibanez B, James S, Agewall S, Antunes MJ, Bucciarelli-Ducci C, Bueno H, Caforio ALP, Crea F, Goudevenos JA, Halvorsen S, Hindricks G, Kastrati A, Lenzen MJ, Prescott E, Roffi M, Valgimigli M, Varenhorst C, Vranckx P, Widimský P, ESC Scientific Document Group (2018). 2017 ESC Guidelines for the management of acute myocardial infarction in patients presenting with ST-segment elevation: the Task Force for the management of acute myocardial infarction in patients presenting with ST-segment elevation of the European Society of Cardiology (ESC). Eur Heart J.

[25] Kettner J, Sramko M, Holek M, Pirk J, Kautzner J (2013). Utility of intra-aortic balloon pump support for ventricular septal rupture and acute mitral regurgitation complicating acute myocardial infarction. Am J Cardiol.

[26] Hsu S, Kambhampati S, Sciortino CM, Russell SD, Schulman SP (2018). Predictors of intra-aortic balloon pump hemodynamic failure in non-acute myocardial infarction cardiogenic shock. Am Heart J.

[27] Gul B, Bellumkonda L (2019). Usefulness of intra-aortic balloon pump in patients with cardiogenic shock. Am J Cardiol.

[28] Fried JA, Nair A, Takeda K, Clerkin K, Topkara VK, Masoumi A (2018). Clinical and hemodynamic effects of intra-aortic balloon pump therapy in chronic heart failure patients with cardiogenic shock. J Heart Lung Transplant.

[29] de Jong MM, Lorusso R, Al Awami F, Matteuci F, Parise O, Lozekoot P (2018). Vascular complications following intra-aortic balloon pump implantation: an updated review. Perfusion..

[30] den Uil CA, Van Mieghem NM, Bastos MB, Jewbali LS, Lenzen MJ, Engstrom AE (2019). Primary intra-aortic balloon support versus inotropes for decompensated heart failure and low output: a randomised trial. EuroIntervention.

[31] Thiele H, Zeymer U, Neumann FJ, Ferenc M, Olbrich HG, Hausleiter J, Richardt G, Hennersdorf M, Empen K, Fuernau G, Desch S, Eitel I, Hambrecht R, Fuhrmann J, Böhm M, Ebelt H, Schneider S, Schuler G, Werdan K, IABP-SHOCK II Trial Investigators (2012). Intraaortic balloon support for myocardial infarction with cardiogenic shock. N Engl J Med.

[32] Umakanthan R, Hoff SJ, Solenkova N, Wigger MA, Keebler ME, Lenneman A, Leacche M, DiSalvo TG, Ooi H, Naftilan AJ, Byrne JG, Ahmad RM (2012). Benefits of ambulatory axillary intra-aortic balloon pump for circulatory support as bridge to heart transplant. J Thorac Cardiovasc Surg.

[33] Helgestad OKL, Josiassen J, Hassager C, Jensen LO, Holmvang L, Udesen NLJ, Schmidt H, Berg Ravn H, Moller JE (2020). Contemporary trends in use of mechanical circulatory support in patients with acute MI and cardiogenic shock. Open Heart.

[34] Vallabhajosyula S, Prasad A, Sandhu GS, Bell MR, Gulati R, Eleid MF, et al. Mechanical circulatory support-assisted early percutaneous coronary intervention in acute myocardial infarction with cardiogenic shock: 10-year national temporal trends, predictors and outcomes. EuroIntervention. 2019;EIJ-D-19-00226. 10.4244/EIJ-D-19-00226 PMC9725008 31746759

[35] Unverzagt S, Buerke M, de Waha A, Haerting J, Pietzner D, Seyfarth M (2015). Intra-aortic balloon pump counterpulsation (IABP) for myocardial infarction complicated by cardiogenic shock. Cochrane Database Syst Rev.

[36] Ponikowski P, Voors AA, Anker SD, Bueno H, Cleland JGF, Coats AJS (2016). 2016 ESC Guidelines for the diagnosis and treatment of acute and chronic heart failure: the Task Force for the diagnosis and treatment of acute and chronic heart failure of the European Society of Cardiology (ESC) developed with the special contribution of the Heart Failure Association (HFA) of the ESC. Eur Heart J.

[37] van Diepen S, Katz JN, Albert NM, Henry TD, Jacobs AK, Kapur NK, Kilic A, Menon V, Ohman EM, Sweitzer NK, Thiele H, Washam JB, Cohen MG, American Heart Association Council on Clinical Cardiology; Council on Cardiovascular and Stroke Nursing; Council on Quality of Care and Outcomes Research; and Mission: Lifeline (2017). Contemporary management of cardiogenic shock: a scientific statement from the American Heart Association. Circulation..

[38] Cui K, Lyu S, Liu H, Song X, Yuan F, Xu F, Zhang M, Zhang M, Wang W, Zhang D, Tian J, Yan Y, Zhou K, Chen L (2019). Timing of initiation of intra-aortic balloon pump in patients with acute myocardial infarction complicated by cardiogenic shock: a meta-analysis. Clin Cardiol.

[39] Pieri M, Sorrentino T, Oppizzi M, Melisurgo G, Lembo R, Colombo A, Zangrillo A, Pappalardo F (2018). The role of different mechanical circulatory support devices and their timing of implantation on myocardial damage and mid-term recovery in acute myocardial infarction related cardiogenic shock. J Interv Cardiol.

[40] Thiele H, Jobs A, Ouweneel DM, Henriques JPS, Seyfarth M, Desch S, Eitel I, Pöss J, Fuernau G, de Waha S (2017). Percutaneous short-term active mechanical support devices in cardiogenic shock: a systematic review and collaborative meta-analysis of randomized trials. Eur Heart J.

[41] Ouweneel DM, Eriksen E, Sjauw KD, van Dongen IM, Hirsch A, Packer EJ (2017). Percutaneous mechanical circulatory support versus intra-aortic balloon pump in cardiogenic shock after acute myocardial infarction. J Am Coll Cardiol.

[42] Schrage B, Ibrahim K, Loehn T, Werner N, Sinning JM, Pappalardo F, Pieri M, Skurk C, Lauten A, Landmesser U, Westenfeld R, Horn P, Pauschinger M, Eckner D, Twerenbold R, Nordbeck P, Salinger T, Abel P, Empen K, Busch MC, Felix SB, Sieweke JT, Møller JE, Pareek N, Hill J, MacCarthy P, Bergmann MW, Henriques JPS, Möbius-Winkler S, Schulze PC, Ouarrak T, Zeymer U, Schneider S, Blankenberg S, Thiele H, Schäfer A, Westermann D (2019). Impella support for acute myocardial infarction complicated by cardiogenic shock. Circulation..

[43] Dhruva SS, Ross JS, Mortazavi BJ, Hurley NC, Krumholz HM, Curtis JP, et al. Association of use of an intravascular microaxial left ventricular assist device vs intra-aortic balloon pump with in-hospital mortality and major bleeding among patients with acute myocardial infarction complicated by cardiogenic shock. JAMA. 2020;e200254. 10.1001/jama.2020.0254 PMC7042879 32040163

[44] Kapur NK, Paruchuri V, Majithia A, Esposito M, Shih H, Weintraub A, Kiernan M, Pham DT, Denofrio D, Kimmelstiel C (2015). Hemodynamic effects of standard versus larger-capacity intraaortic balloon counterpulsation pumps. J Invasive Cardiol.

[45] Visveswaran GK, Cohen M, Seliem A, DiVita M, McNamara JKR, Dave A (2017). A single center tertiary care experience utilizing the large volume mega 50cc intra-aortic balloon counterpulsation in contemporary clinical practice. Catheter Cardiovasc Interv.

[46] Baran DA, Visveswaran GK, Seliem A, DiVita M, Wasty N, Cohen M (2018). Differential responses to larger volume intra-aortic balloon counterpulsation: hemodynamic and clinical outcomes. Catheter Cardiovasc Interv.

[47] Berg DD, Bohula EA, van Diepen S, Katz JN, Alviar CL, Baird-Zars VM, Barnett CF, Barsness GW, Burke JA, Cremer PC, Cruz J, Daniels LB, DeFilippis A, Haleem A, Hollenberg SM, Horowitz JM, Keller N, Kontos MC, Lawler PR, Menon V, Metkus TS, Ng J, Orgel R, Overgaard CB, Park JG, Phreaner N, Roswell RO, Schulman SP, Jeffrey Snell R, Solomon MA, Ternus B, Tymchak W, Vikram F, Morrow DA (2019). Epidemiology of shock in contemporary cardiac intensive care units. Circ Cardiovasc Qual Outcomes.

[48] Schrage B, Weimann J, Dabboura S, Yan I, Hilal R, Becher PM, et al. Patient characteristics, treatment and outcome in non-ischemic vs. ischemic cardiogenic shock. J Clin Med. 2020;9(4):931. 10.3390/jcm9040931 PMC7230560 32231121

[49] Harjola VP, Lassus J, Sionis A, Kober L, Tarvasmaki T, Spinar J (2015). Clinical picture and risk prediction of short-term mortality in cardiogenic shock. Eur J Heart Fail.

[50] Malick W, Fried JA, Masoumi A, Nair A, Zuver A, Huang A (2019). Comparison of the hemodynamic response to intra-aortic balloon counterpulsation in patients with cardiogenic shock resulting from acute myocardial infarction versus acute decompensated heart failure. Am J Cardiol.

[51] Norkiene I, Ringaitiene D, Rucinskas K, Samalavicius R, Baublys A, Miniauskas S, Sirvydis V (2007). Intra-aortic balloon counterpulsation in decompensated cardiomyopathy patients: bridge to transplantation or assist device. Interact Cardiovasc Thorac Surg.

[52] Gjesdal O, Gude E, Arora S, Leivestad T, Andreassen AK, Gullestad L, Aaberge L, Brunvand H, Edvardsen T, Geiran OR, Simonsen S (2009). Intra-aortic balloon counterpulsation as a bridge to heart transplantation does not impair long-term survival. Eur J Heart Fail.

[53] Mizuno M, Sato N, Kajimoto K, Sakata Y, Minami Y, Munakata R, Hagiwara N, Takano T, Acute Decompensated Heart Failure Syndromes [ATTEND] investigators (2014). Intra-aortic balloon counterpulsation for acute decompensated heart failure. Int J Cardiol.

[54] Ntalianis A, Kapelios CJ, Kanakakis J, Repasos E, Pantsios C, Nana E, Kontogiannis C, Malliaras K, Tsamatsoulis M, Kaldara E, Charitos C, Nanas JN (2015). Prolonged intra-aortic balloon pump support in biventricular heart failure induces right ventricular reverse remodeling. Int J Cardiol.

[55] Sintek MA, Gdowski M, Lindman BR, Nassif M, Lavine KJ, Novak E, Bach RG, Silvestry SC, Mann DL, Joseph SM (2015). Intra-aortic balloon counterpulsation in patients with chronic heart failure and cardiogenic shock: clinical response and predictors of stabilization. J Card Fail.

[56] den Uil CA, Galli G, Jewbali LS, Caliskan K, Manintveld OC, Brugts JJ (2017). First-line support by intra-aortic balloon pump in non-ischaemic cardiogenic shock in the era of modern ventricular assist devices. Cardiology..

[57] Morici N, Oliva F, Ajello S, Stucchi M, Sacco A, Cipriani MG, de Bonis M, Garascia A, Gagliardone MP, Melisurgo G, Russo CF, la Vecchia C, Frigerio M, Pappalardo F (2018). Management of cardiogenic shock in acute decompensated chronic heart failure: the ALTSHOCK phase II clinical trial. Am Heart J.

[58] Ouweneel DM, Henriques JP (2012). Percutaneous cardiac support devices for cardiogenic shock: current indications and recommendations. Heart..

[59] den Uil CA, Akin S, Jewbali LS, Dos Reis MD, Brugts JJ, Constantinescu AA (2017). Short-term mechanical circulatory support as a bridge to durable left ventricular assist device implantation in refractory cardiogenic shock: a systematic review and meta-analysis. Eur J Cardiothorac Surg.

[60] Hawranek M, Gierlotka M, Pres D, Zembala M, Gasior M (2018). Nonroutine use of intra-aortic balloon pump in cardiogenic shock complicating myocardial infarction with successful and unsuccessful primary percutaneous coronary intervention. JACC Cardiovasc Interv.

[61] Benjamin EJ, Virani SS, Callaway CW, Chamberlain AM, Chang AR, Cheng S, Chiuve SE, Cushman M, Delling FN, Deo R, de Ferranti SD, Ferguson JF, Fornage M, Gillespie C, Isasi CR, Jiménez MC, Jordan LC, Judd SE, Lackland D, Lichtman JH, Lisabeth L, Liu S, Longenecker CT, Lutsey PL, Mackey JS, Matchar DB, Matsushita K, Mussolino ME, Nasir K, O’Flaherty M, Palaniappan LP, Pandey A, Pandey DK, Reeves MJ, Ritchey MD, Rodriguez CJ, Roth GA, Rosamond WD, Sampson UKA, Satou GM, Shah SH, Spartano NL, Tirschwell DL, Tsao CW, Voeks JH, Willey JZ, Wilkins JT, Wu JH, Alger HM, Wong SS, Muntner P, American Heart Association Council on Epidemiology and Prevention Statistics Committee and Stroke Statistics Subcommittee (2018). Heart disease and stroke statistics-2018 update: a report from the American Heart Association. Circulation..

[62] Prondzinsky R, Unverzagt S, Russ M, Lemm H, Swyter M, Wegener N, Buerke U, Raaz U, Ebelt H, Schlitt A, Heinroth K, Haerting J, Werdan K, Buerke M (2012). Hemodynamic effects of intra-aortic balloon counterpulsation in patients with acute myocardial infarction complicated by cardiogenic shock: the prospective, randomized IABP shock trial. Shock..

[63] Thiele H, Sick P, Boudriot E, Diederich KW, Hambrecht R, Niebauer J, Schuler G (2005). Randomized comparison of intra-aortic balloon support with a percutaneous left ventricular assist device in patients with revascularized acute myocardial infarction complicated by cardiogenic shock. Eur Heart J.

[64] Seyfarth M, Sibbing D, Bauer I, Frohlich G, Bott-Flugel L, Byrne R (2008). A randomized clinical trial to evaluate the safety and efficacy of a percutaneous left ventricular assist device versus intra-aortic balloon pumping for treatment of cardiogenic shock caused by myocardial infarction. J Am Coll Cardiol.

[65] Kapur NK, Hirst CS (2018). Counterpulsation requires pulsation: IABP use in patients with heart failure without acute MI. Catheter Cardiovasc Interv.

[66] Lauten P, Rademacher W, Goebel B, Kretzschmar D, Figulla HR, Lauten A, Ferrari M (2012). Intra-aortic counterpulsation for hemodynamic support in patients with acute ischemic versus non-ischemic heart failure. J Invasive Cardiol.

[67] Ton VK, Xie R, Hernandez-Montfort JA, Meyns B, Nakatani T, Yanase M (2020). Short- and long-term adverse events in patients on temporary circulatory support before durable ventricular assist device: An IMACS registry analysis. J Heart Lung Transplant.

[68] Krishnamoorthy A, DeVore AD, Sun JL, Barnett AS, Samsky MD, Shaw LK (2017). The impact of a failing right heart in patients supported by intra-aortic balloon counterpulsation. Eur Heart J Acute Cardiovasc Care.

[69] Francis GS, Bartos JA, Adatya S (2014). Inotropes. J Am Coll Cardiol.

[70] Abraham WT, Adams KF, Fonarow GC, Costanzo MR, Berkowitz RL, LeJemtel TH, Cheng ML, Wynne J, ADHERE Scientific Advisory Committee and Investigators, ADHERE Study Group (2005). In-hospital mortality in patients with acute decompensated heart failure requiring intravenous vasoactive medications: an analysis from the Acute Decompensated Heart Failure National Registry (ADHERE). J Am Coll Cardiol.

